# Physician and Medical Student Burnout, a Narrative Literature Review: Challenges, Strategies, and a Call to Action

**DOI:** 10.3390/jcm14072263

**Published:** 2025-03-26

**Authors:** Santiago Cotobal Rodeles, Francisco Javier Martín Sánchez, Manuel Martínez-Sellés

**Affiliations:** 1 Unidad de Geriatría, Complejo Asistencial Universitario, 37007 Salamanca, Spain; scotobalr@gmail.com; 2 Hospital Enfermera Isabel Zendal, 28055 Madrid, Spain; fjjms@hotmail.com; 3 Department of Cardiology, Hospital Universitario Gregorio Marañón, Instituto de Investigación Sanitaria Gregorio Marañon, Calle Doctor Esquerdo, 46, 28007 Madrid, Spain; 4 School of Medicine, Universidad Complutense de Madrid, 28040 Madrid, Spain; 5 School of Health and Biomedical Sciences, Universidad Europea de Madrid, 28670 Madrid, Spain; 6 Centro de Investigación Biomédica en Red—Enfermedades Cardiovasculares (CIBERCV), Instituto de Salud Carlos III, C/Monforte de Lemos 3-5, Pabellón 11, Planta 0, 28029 Madrid, Spain

**Keywords:** burnout, healthcare, depression, resilience

## Abstract

**Background**: Burnout is a state of emotional, physical, and mental exhaustion produced by excessive and prolonged professional stress. Its prevalence is unclear, and figures from 2 to 81% have been reported, although studies focused on this issue are scarce and inconsistent definitions and the absence of validated measurement tools make comparisons difficult. **Methods**: Our narrative review’s purpose was to explore physician and medical student burnout across medical specialties and in specific subgroups, including young doctors, researchers, and female physicians. We also assess burnout effects in medical students and patients and the possible strategies to prevent and reverse it. **Results**: Burnout affects doctors, medical students, and patients. It impacts significantly on physicians mental health and can be the trigger for depression, substance abuse, and suicide attempts. Moreover, this psychological and physical exhaustion can also increase the risk of systemic conditions such as cardiovascular disease. Physician burnout increases the risk of medical errors, reduces professional efficacy, and might compromise patients’ safety. Strategies focusing on mental, physical, social, and occupational well-being can help to prevent and treat burnout. These include resilience training, self-care, exercise, work–life balance, and institutional changes, such as reducing administrative burdens and improving electronic health record systems. Medical students’ burnout might be triggered by specific problems related to their young age, economic situation, exam stress and workload, high academic expectations, lack of support, and others. **Conclusions**: Burnout is common in physicians and medical students, negatively affecting mental health, professional/academic efficacy, and patient outcomes. Addressing burnout requires a multifaceted approach, including individual strategies and systemic changes within institutions.

## 1. Introduction, Diagnosis and Assessment

The 11th revision of the International Classification of Diseases defines burnout as a syndrome resulting from chronic workplace stress that has not been successfully managed [1]. The World Health Organization recognizes burnout as an occupational hazard resulting from unmanaged chronic stress, with three dimensions: exhaustion, increased mental distance or cynicism regarding the job, and reduced professional efficacy [1]. The differentiation of professional stress-related depression, chronic tiredness, and burnout is unclear [2,3].

Our objective was to explore physician and medical student burnout across medical specialties and in specific subgroups, including young doctors, researchers, and female physicians. This article will cover the following burnout items and their relevance to clinical practice: (1) diagnosis and assessment, (2) prevalence and risk factors, (3) consequences, (4) prevention and treatment, and (5) human flourishing. We will finish with the study of burnout in medical students.

Regarding burnout diagnosis, several tools have been developed to assess and measure the degree of burnout (Table 1); the main three are as follows: (1) The Maslach Burnout Inventory—Human Services Survey instrument [4], with 22 seven-point items covering emotional exhaustion, depersonalization, and personal accomplishment. The answers are quantified from 0 = never to 6 = every day [5], but do not differentiate if symptoms are related to work or not [6]. An abbreviated scale is available but seems to have lower accuracy [5,7,8,9,10]. (2) The Stanford Physician Wellness survey, which measures burnout symptoms including emotional exhaustion and interpersonal disengagement, professional fulfillment, self-compassion, perceived appreciation from others, perceived support from peers, mission alignment, meaningfulness of clinical care, control over schedule, a brain-health nutrition assessment, mindfulness, job-related damage to personal relationships, and self-reported medical errors [11,12]. (3) The Mini-Z Work–Life and Burnout Reduction Instrument, a 10-question instrument that provides information on satisfaction, burnout, and remediable work conditions, with subscales for medical residents such as the Mini ReZ, with 5 additional questions [13,14]. In addition, wearables and biosensors can also be used to detect burnout-related symptoms, detecting, for instance, low step count, time in bed, heart rate, and heart rate variability [15].

Burnout has a strong impact on physicians [4,16], and is both a cause and consequence of a non-humanistic approach [6]. Doctors tend to impose responsibilities on themselves that are frequently above their ability to use time wisely so as to enjoy their life and balance their ambitions [17]. Different risk factors for physician burnout have been described.

Most burnout occurs in physicians without pre-existing psychological conditions, but pre-existing psychiatric and psychological disorders can contribute to burnout. Culture- and work-related issues also have a strong influence [18,19]. In addition, the COVID-19 pandemic seems to have been a catalyst of burnout, intensifying workplace stress and dehumanization [20].

Our narrative review aims to synthesize recent knowledge regarding physician burnout in different medical specialties and subgroups. We also assess burnout effects in physicians and patients and the possible strategies to prevent and reverse it.

## 2. Prevalence and Risk Factors

**Figure 1:**
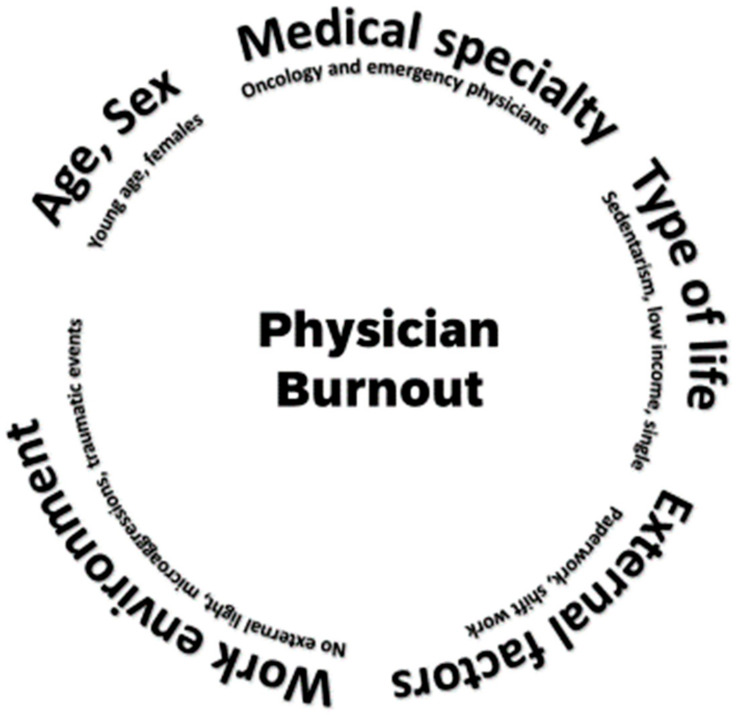
The main factors that influence the prevalence of physician burnout.

Burnout is a common occupational hazard among physicians [2]. Its prevalence is unclear, and figures from 2 to 81% have been reported [21]. This is largely due to the different diagnostic criteria, definitions, and assessment methods used [9,21,22,23]. In some cases, even in the same sample, the rates can change from 13% to 69% according to the criteria used [24]. In addition, geographical variations have been described [2] and the prevalence is influenced by several factors (Figure 1), such as degree of responsibility, role, sex, age, and the type of medical specialty (Table 2) [7,25,26,27]. Oncology [28,29] and emergency physicians seem to be at a particularly high risk [30,31]. This also seems to be the case with female physicians [2,6,32,33]. Other risk factors are physical inactivity [34], young/middle age [8], low incomes, and being single [6,27,33]. External factors also have an important role, such as excessive paperwork [27,35], the overuse of electronic health records [12,36], shift work [37], work environments without external light [7,25], microaggressions [38], and traumatic events [30]. Most risk factors are similar to the ones described for depression and suicide [10,13,22,27,35,39].

Burnout seems to be particularly frequent in young doctors. In a recent Medscape survey, almost half of generation X (40–54 years old) physicians report burnout compared with 38% of millennials (25–39 years old) and 39% of baby boomers (55–73 years old). Older physicians may have higher levels of resilience and be able to balance their personal lives with their occupation as well as possibly engaging in certain behaviors that increase social support. On the other hand, residency might be a challenge where personal/family and professional roles can come into conflict [40]. High frequencies of depression and suicidal ideation have been documented in medical students [41,42], and burnout is more prevalent in residents and early-career physicians than among their older peers [43]. In addition, work–life balance is frequently seen as a major challenge, and few consider the option of starting a family during residency [44,45]. In fact, specific interventions to promote emotional health and resilience in young physicians have been recommended [46].

Burnout is more common in female physicians [2,6,32,33], and may be up to 60% more frequent than in males [47]. This is due to multiple reasons, including some sex-related risk factors, such as unequal pay, a lack of mentorship, limited leadership opportunities, time constraints, increased household responsibilities or childcare, and increased maternal age [48]. In addition, burnout seems to be triggered differently according to sex; depersonalization is usually the key factor in males as compared to emotional exhaustion in females [49].

Burnout is also more frequent in academic physicians. Clinical scientists have three major missions (education, patient care, and research), and all of them can be a cause of burnout [50]. Most studies focused on medical burnout have been performed in Western countries; in any case, the figures in low- and middle-income countries seem to be similar or even higher [51].

## 3. Consequences

Burnout has consequences on physicians, patients, healthcare systems, education, and biomedical research. [50,52,53]. Several conditions and symptoms have been associated with burnout, including cardiovascular risk factors (hypercholesterolemia, diabetes, hypertension) and conditions (coronary artery disease, death at a young age) [33], musculoskeletal conditions (pain, fatigue), and others (headaches, gastrointestinal issues, increased cortisol levels). Moreover, emotional exhaustion, depersonalization, and a reduced sense of personal accomplishment increase maladaptive behaviors, eating disorders, substances misuse, sleep disturbances, depression, motor vehicles crashes, and increase suicide risks [2,52,54,55,56], even in physicians without clear depression [57].

Regarding patients and the healthcare system, burnout decreases healthcare quality, increases the risk of medical errors and the odds of being named in a medical malpractice suit, and is associated with longer recovery times and lower patient satisfaction [2,32,52]. Burnout decreases productivity and is associated with early physician retirement and increased costs. The estimated cost to replace a single physician might range from hundreds of thousands to well over one million USD [52,58].

## 4. Prevention and Treatment

To prevent and treat physician burnout, a comprehensive approach with institutional/systemic improvements and local/personal interventions is needed [4,50] (Table 3). Strategies should focus on mental, physical, social, and occupational well-being [59]. Mental well-being-focused interventions have proven to be effective [32], improving resilience by providing emotional support resources such as counseling services, peer support groups, or mind retreats [4,18,25,32,60,61,62,63]. Programs aimed at improving physical well-being might recommend dietary lifestyle changes, exercise, stretching techniques, ergonomic postures, sleep hygiene, and reducing alcohol and caffeine consumption [4]. Regarding social well-being, it is important to achieve an adequate work–life balance [39,63]. For instance, spending at least 20% of our time on fulfilling activities is associated with decreased burnout and increased well-being [64]. Social support networks of physicians can also have a protective effect [65]. Occupational well-being frequently needs an institutional approach, ensuring enough staff, reducing red tape and nonclinical responsibilities [18,25,32,63], improving electronic health records [26], and implementing automated dictation [2,12]. Programs such as Train The Trainers [2,60] or web-based professional group coaching are also good options [30,54,66,67]. However, some physicians may perceive these interventions as an added workload. Unfortunately, this vision might make it so that some institutions do not implement these changes or end up blaming the victims [20,68].

Burnout has been examined in the following specific subgroups.

Young physicians, particularly those in training or early-career stages, often experience burnout due to long working hours, high levels of responsibility, and limited autonomy [40]. To mitigate these challenges, institutions can implement structured mentorship programs that provide guidance, emotional support, and career development advice. Adjusting schedules to allow for adequate rest and work–life balance is critical, as is ensuring access to mental health resources without stigma [46]. Teaching time management and stress management skills during medical residence can also prepare young physicians for the demands of their roles. Furthermore, involving young doctors in decision-making processes can enhance their sense of agency and reduce feelings of helplessness in high-pressure environments.

Female physicians face burnout more frequently than males [2,6,32,33,43] due to additional stressors, such as gender biases, work–life conflicts, and the disproportionate burden of caregiving responsibilities outside of work [48]. Creating policies that support flexible working hours and parental leave can help alleviate these pressures. Promoting gender equity in leadership opportunities and addressing workplace discrimination and harassment are essential steps in fostering an inclusive and supportive environment. Additionally, offering networking opportunities and peer support groups specifically for female physicians can help combat isolation and provide a platform to share experiences and solutions.

Academic physicians and clinical scientists add to their clinical workload with research and teaching. Compared to non-academic physicians, medical doctors with scientific and academic responsibilities are more prone to burnout [50]. Tailoring interventions for this subgroup should focus on systemic changes that make science compatible with patient care without having a negative impact on scientific physicians’ personal lives.

## 5. Human Flourishing

It is not easy to define the opposite of burnout, but human flourishing might be a good option [69], as flourishing indices are associated with well-being and inversely related to burnout metrics [70]. Human flourishing depends on doing or being well in five broad domains: (i) happiness and life satisfaction; (ii) health, both mental and physical; (iii) purpose; (iv) virtue; and (v) close social relationships. Like resilience, flourishing is inversely associated with burnout [71], and this inverse association is particularly strong in the case of flourishing. Flourishing can be assessed with standardized questions regarding multi-dimensional and complete well-being [72]. At least some of these factors might be associated with medical practice. Flourishing measures mental and physical health, well-being, purpose, engagement, and relationships. In the context of active physicians, flourishing can manifest as a sense of professional fulfillment, personal growth, and resilience, despite the stresses of the medical field. Doctors’ satisfaction/happiness have been reported to be associated with love/relationships/family [71] and religion [73,74]. The possible correlation between religion/spirituality and flourishing is relevant, as both could be shields against burnout. Physicians often experience high levels of stress and anxiety due to several factors, such as long work hours, sleep deprivation, high expectations, and the emotional toll of patient care. Amidst these challenges, religion and spirituality could have the potential to foster resilience and flourishing, decreasing the risk of burnout by offering solace and perspective. Supporting physicians spiritual well-being as part of a holistic approach might increase their flourishing levels and protect them against burnout. Programs might consider incorporating spirituality-oriented interventions and, when appropriate, facilitate religious practice, to help physicians cope with the demands of their profession. Regarding family, it should be noted that reconciling professional success with a fulfilling and satisfying personal and familiar life is possible and there is arguably a need to remind physicians that our work should not be the only priority in our life [40]. Finally, new strategies such as professional coaching might have the potential of improving physician well-being and flourishing [75]. We need to acknowledge that physician burnout is a significant problem in the medical profession, associated with depression, anxiety [76], and suicidality [55]. However, burnout is reversible and preventable [77]. Interventions, including but not limited to group interventions, relaxation and assertiveness training, facilitated discussion groups, and promoting a healthy work environment, can be used to promote flourishing and reduce burnout.

## 6. Medical Students

Burnout has also become a prevalent concern among medical students [78,79] and other healthcare students, such as nursing students [80]. The prevalence of burnout syndrome in medical students is quite heterogeneous; in some studies it can be as high as 88% [81], but a more realistic figure is about 50% [82]. This rate, based on a recent national survey of Israeli medical students using the Maslach Burnout Inventory—Student Survey, means that half of medical students have burnout syndrome, stressing the need of measures to change this situation. Even early-year medical students already have symptoms and signs of burnout, including exhaustion, a lack of motivation, and changes in personality [83], and a gradual increase in cynicism during medical education has been described [84]. Moreover, the rate of burnout seems to increase as months pass during the academic year [85]. Medical students have a desire to gain a greater awareness of burnout and insight into preventative strategies within their curriculum [83]. Burnout affects mental health, increasing risks of depression and suicide, at least in part due to rigorous training demands. Comparative research between medical and non-medical students is limited, but, compared to other students, medical students seem to have higher suicide risks, depression, perfectionism, burnout, and loneliness [86]. In addition to mental health, academic performance and future professional practice can be impaired due to burnout. Some of the causes, consequences, and potential strategies to address burnout among medical students are similar to the ones we have previously addressed for physicians. However, in other cases, burnout might be triggered by specific problems related to students’ young age, economic situation, exams stress [87] and workload, high academic expectations, lack of support, and other factors. Understanding and addressing burnout is crucial for promoting the well-being of medical students and ensuring the sustainability of the future elite healthcare workforce.

Among medical students, burnout has been recognized as a significant issue, with rates that exceed those found in the general student population. The demanding nature of medical education, characterized by long hours of study, high-stakes examinations, first patient contact, and intensive clinical training, contributes to this phenomenon. As the next generation of physicians, the well-being of medical students is crucial for the effective delivery of patient care. A burned-out medical student has every chance of becoming a burned-out physician. Understanding the factors that lead to burnout and implementing strategies in medical schools to address them is vital for fostering a healthier learning environment.

Table 4 depicts specific causes of burnout in medical students.

Academic pressure is probably the main trigger of medical students’ burnout [88]. The rigorous nature of medical curricula demands significant time and cognitive investment that might lead to chronic stress. The constant need to achieve high grades and excel in standardized tests can foster a perfectionistic mindset, making students susceptible to burnout. Workload and time constraints are also very common, the heavy workload is a big issue, and balancing lectures, lab work, and clinical rotations might be very difficult. This workload, coupled with time constraints, leaves little room for rest and self-care. In addition, the intense schedule can disrupt sleep patterns and limit opportunities for relaxation and social interactions. Emotional strain during the process of learning to care for patients and witnessing suffering is almost universal and, mainly during the first years, can be emotionally taxing. Students are frequently exposed to different types of human suffering, disease, death, and complex ethical dilemmas. This exposure can contribute to emotional exhaustion and feelings of inadequacy. The lack of support systems is also a problem. Medical students might not have adequate mentorship, and this can exacerbate feelings of isolation and stress. Moreover, the competitive nature of medical education can discourage students from seeking help, fearing that it might be perceived as a sign of weakness. Finally, although the situations might be different according to the country and academic system, financial pressures can increase the previously mentioned situations. The high cost of medical education often results in significant debt, adding financial stress to the already heavy emotional burden. Concerns about future job prospects and the ability to repay loans can further contribute to anxiety and burnout.

Being away from home is associated with burnout in medical students, as is also the case for physicians in training who must change domicile for their rotations. This is particularly relevant for those following medical studies abroad, who suffer the combination of academic stress, the stress derived from their new living situation, and, in several cases, the stress of using a language that is not their native tongue [89].

Intergenerational tensions seem to be common, and assertions that medical students or young physicians are not as dedicated to medicine and implicit assumptions that later-career physicians should retire are frequent [90]. Strong work identity and tensions between different generations may trigger burnout and make it necessary to reconsider the ways we examine relations between work identity and age.

The specific consequences of burnout in medical students include decreased academic performance due to impaired concentration, reduced motivation, and cognitive difficulties, leading to lower marks, delays in course completion, and higher dropout rates. In recent years, in medical schools, burnout has been shown to be able to impair clinical decision-making, empathy, and communication skills, leading to a decreased ability to provide compassionate care. In addition, the mental health issues are similar to the ones we have described for physicians, as student burnout is also closely linked to mental health disorders such as depression, anxiety, and suicidal ideation.

Some factors associated with the risk of burnout syndrome in medical students are modifiable [91]. Different strategies for preventing burnout have been proposed. Probably, more than a unique approach, addressing burnout requires a multifaceted plan, involving both individual-level and institutional strategies. Table 5 shows interventions that should be included in a burnout prevention program in medical schools.

The promotion of resilience and well-being can be achieved with programs focused on building resilience, such as mindfulness training [92], promoting spirituality, stress management workshops, muscle relaxation, behavioral therapy, recreational music [80], and cognitive–behavioral strategies. Encouraging students to maintain a balanced lifestyle, with time for exercise, hobbies, and social connections, can also improve their overall well-being and decrease burnout risk. Fostering a supportive environment can play a crucial role by creating a culture that prioritizes student well-being. This includes providing access to mental health services, mentorship programs, and peer support networks. In fact, a supportive environment encourages students to seek help when needed without fear of stigma. Curriculum reform is probably the best institutional way to produce a change. Reducing the academic load and offering greater flexibility in the curriculum can help alleviate some of the pressure on students. Incorporating more opportunities for self-directed learning, avoiding unnecessary memorization, and providing protected time for rest and disconnection can make a significant difference. Developing curricula that improve medical students’ resilience through applying self-care techniques in stressful situations may reduce burnout [93]. Addressing financial concerns may be extremely important in some countries. Offering scholarships, reducing tuition costs, and providing financial planning resources can help alleviate the financial burden on medical students. This can reduce anxiety related to debt and future income, allowing students to focus more on their studies and personal well-being. Encouraging work–life balance, in a similar way to that which we have presented for physicians, helps students to understand the importance of work–life balance, and modeling this behavior within academic and clinical settings can prevent the normalization of overworking. Promoting social support and a healthy lifestyle with physical activity [94] among medical students has been associated with a reduction in burnout risk [85]. Faculty and mentors who emphasize the value of taking time off for personal activities can set a positive example for students. Student-driven feedback and survey results can help to prompt medical schools to develop more robust mental healthcare models and drive much-needed structural changes that reduce burnout risk [78]. Finally, emphasizing empathy [95], in particular cognitive empathy, is an excellent way to prevent burnout in medical students [96], which is probably related to cognitive empathy’s positive association with personal accomplishment. This is also the case with reflection-based interventions [97].

Burned-out medical students may become burned-out doctors. The early identification of those prone to burnout is essential to the implementation of prompt programs aimed at its treatment and prevention. Screening tools as the Medical Student Well-Being Index (MSWBI) or the Maslach Burnout Inventory—Student Survey (MBI-SS) can serve as brief assessment tools to identify medical students in severe psychological distress [98,99]. In addition, these tools can be used to assess wellness initiatives effectiveness at reducing burnout.

## 7. Cost

The growing prevalence of physician burnout has prompted increased scrutiny into its economic consequences, which are still largely unknown. Burnout is linked to increased rates of physician turnover, reduced clinical productivity, and medical errors. These factors are associated with higher healthcare costs. Addressing the financial burden of burnout is essential for policymakers, healthcare administrators, and medical institutions aiming to improve physician well-being and healthcare system sustainability. Burnout is a leading cause of physician turnover, with many practitioners leaving their jobs due to excessive stress, dissatisfaction, and emotional fatigue. Replacing a single physician has costs that are variable among countries and regions, considering recruitment, onboarding, and lost productivity during the transition period. A national study performed in the United States estimated approximately USD 5 billion in costs related to physician turnover and reduced clinical hours attributable to burnout each year, with an annual economic cost of approximately USD 7600 per employed physician each year [100]. The excess healthcare expenditures attributable to primary care physician turnover in the United States is about USD 979 million, with USD 260 million specifically attributable to burnout-related turnover [101].

In addition, burnout leads to lower efficiency and effectiveness, resulting in fewer patient visits and delayed decision-making. A burned-out physician may contribute to increased patient wait times and diminished healthcare access. Physician burnout is correlated with a higher incidence of medical errors [10,102], which can lead to malpractice claims and legal settlements. Burned-out physicians are more likely to engage in suboptimal decision-making, leading to unnecessary tests, hospitalizations, and complications. Poor patient outcomes drive up healthcare spending, burdening insurance systems and increasing overall expenditure. Burnout contributes to higher rates of absenteeism, sick leave, and disability claims. As previous evidence suggests that burnout can effectively be reduced with moderate levels of investment, a substantial economic value seems to exist for policy and organizational expenditures aimed at burnout reduction.

## 8. Knowledge Gaps, Recent Research Subtopics, and Future Research Directions

### 8.1. Limitations and Knowledge Gaps

Burnout among physicians and medical students is common and represents a significant concern, yet knowledge gaps persist in understanding its complex causes and consequences [103]. The emotional exhaustion, depersonalization, and reduced personal accomplishment that characterize burnout are well documented; however, the interplay between individual vulnerabilities and systemic, social, and established factors remains underexplored. For instance, the impact of specific institutional policies, cultural stigmas around seeking help, and variations in burnout prevalence across some medical specialties lack comprehensive studies. Additionally, the long-term implications of burnout on career longevity, patient care outcomes, and healthcare system efficiency are inadequately understood, creating challenges for developing targeted interventions [104].

### 8.2. Recent Research Subtopics

Recent studies on burnout have diversified into subtopics that address both prevention and mitigation. Emerging research is also focusing on excessive administrative burdens, which contribute to burnout. Moreover, the recent COVID-19 pandemic highlighted how crises exacerbate stress and strain among healthcare professionals and can be a real burnout catalyst [105].

There is also a growing emphasis on equity, examining how burnout rates differ by sex, socioeconomic status, and ethnicity within medical communities. Data regarding the role of ethnicity in physician burnout are scarce, but recent data from the United States suggest that Black and Indigenous individuals and People of Color have higher rates of poor sleep quality, insomnia, and burnout [106]. Also, in the case of medical students, Asian and Black or African American students seem to have higher risks of burnout [107]. In addition, recent data suggest that, among academic physicians and trainees, sexual and gender minorities have higher levels of burnout and lower levels of professional fulfillment [108].

The role of occupational health in the detection and treatment of physician burnout has been scarcely analyzed, but a low threshold for screening has been recently suggested to identify individuals and to provide them with direct tertiary support [109]. Interestingly, burnout rates seem to also be high among occupational physicians [110]. In the case of general practitioners, they should be an integral part of any plans for rehabilitation and the prevention of burnout, as trust, continuity of care, and attention to the individual are key to fight burnout and are the essence of primary care [111].

### 8.3. Future Research Directions

Future research should prioritize the development of longitudinal studies to better understand the progression of burnout in physicians, medical students, and patients. These studies could provide information regarding burnout effects over time. In addition, exploring the efficacy of organizational changes, such as workload adjustments, flexible scheduling, and institutional wellness programs, will be crucial in creating sustainable solutions. Clinical trials in this setting are difficult, but not impossible, to perform. Additionally, integrating advanced technologies like artificial intelligence to reduce administrative tasks and improve workflow efficiency could be a promising area of inquiry. Artificial intelligence has a clear role in administrative and cognitive burden reduction and could contribute to reducing burnout through innovative solutions such as digital scribes, automated billing, and advanced data management systems [112]. The internet, social media, and technostress can also be a source of physician burnout [113], as stressors related to the use of information and communication technology outside of working hours also contribute to physician burnout.

Studies focused on burnout-resistant physicians would also be welcome. Some physicians seem to avoid burnout, even in high-pressure jobs. Burnout escapees certainly exist, and no one is immune to stress, but some healthcare professionals seem to be immune to burnout. Self-awareness is central, and understanding yourself and what makes you more vulnerable based on personality, temperament, and what you want out of work might help to avoid burnout. Self-regulation tools also help to prevent negative thoughts, emotions, and reactions. However, although individual resistance to problematic professional values is important, it should be complemented by an institutional commitment to creating a culture of compassion for patients and physicians alike [114].

**Figure 2:**
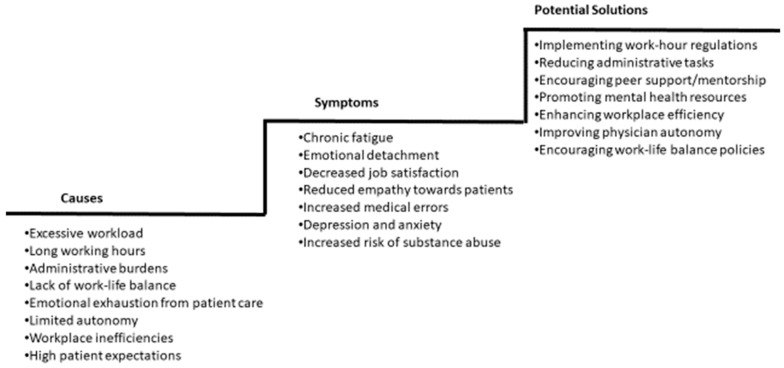
Step-by-step approach to addressing physician burnout.

Research should also expand to include transnational initiatives and global perspectives, comparing how different countries, regions, and healthcare systems address burnout. Initiatives like the European Working Time Directive are welcome. The European Working Time Directive is a European Union initiative to prevent employers from requiring their workforce to work excessively long hours, with implications for health and safety. It requires the working week to be an average of 48 h, with further rights relating to break periods and holiday allowance, such as 11 h of rest a day, a day off each week, a rest break if the working day is longer than 6 hours, and 5.6 weeks paid leave each year. However, most physicians do not seem to perceive a benefit after its implementation [115], and some even suggest that it may contribute to fatigue [116] due to fewer staff being in hospitals at any one time, which leads to the remaining staff being overstretched. Lastly, more studies are needed to evaluate the impact of integrative approaches, combining individual resilience strategies with systemic reforms, to address burnout comprehensively. A systematic review and meta-analysis performed 10 years ago suggested that both individual-focused and structural or organizational strategies can result in clinically meaningful reductions in burnout among physicians [117]. However, further research is needed to establish which interventions are most effective in specific populations, particularly in vulnerable subjects. Recent data suggest that multimodal interventions should be assessed with pre- and post-intervention studies, and that the impact of well-being interventions might be smaller than previously thought [118]. Figure 2 proposes a step-by-step approach to address physician burnout, and Table 6 presents a comprehensive plan for addressing physician burnout.

## 9. Conclusions

Burnout affects doctors, medical students, and patients. Despite the inconsistencies in its definition and diagnosis, its prevalence seems to be high, particularly in some subgroups such as young physicians and women. Comprehensive and coordinated efforts are essential to effectively prevent and treat burnout in order to improve physician and medical student well-being and patient care. Burnout is a critical issue that can have lasting effects on mental health, clinical and academic performance, and professional and personal lives. By understanding the causes and consequences of burnout, institutions and educators can implement strategies to create a more supportive and healthy learning and working environment.

## Figures and Tables

**Table 1:** Main tools to assess and try to quantify burnout.

Burnout Tool	Spheres	Questions	References
Maslach Burnout Inventory—Human Services Survey	Emotional exhaustion	9	[4,5,6]
	Depersonalization	5	
	Personal accomplishment	8	
Maslach Burnout abbreviated scale	Emotional exhaustion	3-4	[5,7,8,9,10]
	Depersonalization	2-3	
	Personal accomplishment	3-4	
Stanford Physician Wellness survey	Culture of wellness	5	[11]
	Efficiency of practice	2	
	Personal resilience	3	
Mini-Z Work–Life	Supportive work environment	5	[14]
	Workplace and electronic health record stress	5	
Mini ReZ	Supportive work environment	5	[14]
	Workplace and electronic health record stress	5	
	Resident experience	5	
Wearables and biosensors	Low step count	-	[15]
	Time in bed		
	Heart rate		
	Heart rate variability		

**Table 2:** Prevalence of burnout according to medical specialty type.

Medical Specialty	Burnout	References
Radiology	62–88%	[24]
Interventional cardiology	69%	[23]
Ophthalmology residents	41%	[25]
Neurosurgery residents	11–67%	[25]
Otorhinolaryngology residents	35–86%	[25]
Plastic surgery residents	58–66%	[25]
Urology residents	38–68%	[25]
Orthopedic surgeons	31–56%	[25]
Emergency physicians	19–86%	[28,29]

**Table 3:** Strategies to prevent and treat burnout.

Sphere	Strategy	References
Mental Well-Being	Counseling service Peer support groups Mind retreats (meditation, mindfulness, tutoring) Spirituality support	[4,18,25,32,60,61,62,63]
Physical Well-Being	Dietary lifestyle changes Exercise Stretching techniques Ergonomic postures Sleep hygiene Reducing alcohol and caffeine consumption	[23]
Social Well-Being	Correct work–life balance Spend time on meaningful and fulfilling activities Social support networks	[39,63,64,65]
Occupational Well-Being	Ensuring enough staff Reducing red tape and nonclinical responsibilities Improving electronic health records Implementing automated dictation Train The Trainers programs Web-based professional group coaching	[2,12,18,25,26,32,60,63,66,67]

**Table 4:** Specific causes of burnout in medical students.

Cause	Reasons
Academic Pressure	Time and cognitive investment Chronic stress Perfectionistic mindset
Workload and Time Constraints	Difficult to balance lectures, lab work, and clinical rotations Little room for rest and self-care Sleep patterns disruption Limited opportunities for relaxation and social interaction
Emotional Strain	Witnessing suffering Exposure to human suffering, death, and complex ethical dilemmas
Lack of Support Systems	No or inadequate mentorship Feelings of isolation and stress Competitive education discourages seeking help
Financial Pressures	High cost of medical education Significant debt and financial stress Concerns about job prospects and ability to repay loans

**Table 5:** The interventions that should be included in a burnout prevention program in medical schools.

Intervention	Explanation
Promoting Resilience and Well-Being	Programs focused on building resilience Encouraging balanced lifestyle Time for exercise, hobbies, and social connections
Fostering a Supportive Environment	Culture that prioritizes student well-being Mental health services, mentorship programs, and peer support networks Encouraging seeking help without fear of stigma
Curriculum Reform	Reducing academic load Flexibility in curricula Self-directed learning Reducing unnecessary memorization Protected time for rest and disconnection
Addressing Financial Concerns	Scholarships Reduced tuition costs Financial planning
Encouraging Work–Life Balance	Present importance of work–life balance Modeling behavior in academic and clinical settings; emphasize value of taking time off Promote social support and healthy lifestyle
Student-Driven Feedback	Survey results Feedback to develop robust models
Promoting Empathy and Reflection	Increase cognitive empathy Emphasize empathy’s association with personal accomplishment Reflection-based intervention**s**

**Table 6:** Comprehensive plan for addressing physician burnout.

Screening	- Validated scores: Maslach Burnout Inventory, Copenhagen Burnout Inventory, Professional Fulfillment Index, Stanford Physician Wellness, Mini-Z Work–Life, Mini ReZ - Self-assessment questionnaires: Periodic anonymous surveys with open-ended questions; digital apps for self-monitoring emotional well-being - Institutional protocols: Routine assessments every 6 months for all physicians; integration of burnout screening into annual employee health checkups - Active search for self and peers early indicators: emotional exhaustion and chronic fatigue; cynicism and detachment from patients and colleagues; reduced sense of professional accomplishment; increased medical errors or decreased efficiency; frequent absenteeism or intent to leave the profession - Screening for physical symptoms related to chronic stress, such as sleep disorders
Preventing, Organizational-Level	- Workload management: Implement fair shift scheduling, reduce excessive work hours, and ensure adequate staffing to prevent overburdening - Administrative reforms: Reduce bureaucratic tasks with scribes and AI-assisted documentation tools - Supportive work environment: Peer-support programs; promote open communication between physicians and leadership; encourage professional autonomy in decision-making - Work–life balance: Breaks and vacations, flexible work schedules, and remote consultation options - Regularly update institutional policies: Maintain a proactive culture of well-being
Preventing, Individual-Level	- Stress management: Resilience training programs, time management, and delegation workshops - Mental support: Confidential counseling services, professional coaches, or wellness advisors - Culture of recognition: Regular positive feedback and appreciation programs; mentorship and career development opportunities
Monitoring	- Regular surveys and biannual anonymous well-being assessments, including feedback on job satisfaction and stressors - Track absenteeism, sick leave, and attrition rates - Identify high-risk areas, departments, and specialties with frequent burnout reports - Structured monthly meetings for open discussions - Confidential suggestion boxes for reporting concerns - Set Key Performance Indicators for well-being - Report findings to leadership for strategic interventions
Diagnosing	- Differentiate burnout from clinical depression and non-job-related anxiety disorders - One-on-one evaluations with mental health professionals - Stress and resilience testing - Identify specific job-related stressors
Follow-up	- Personalized recovery plans: Cognitive–behavioral therapy, career coaching - Temporary work modifications: Reduced clinical hours or workload adjustments, sabbaticals, or mental health leave policies - Wellness and resilience programs: Structured self-care training (sleep hygiene, exercise, nutrition); peer mentorship for ongoing support - Regular follow-up assessments: Gradual reintegration into full workload with supervision; continued access to well-being resources and check-ins
